# Association mapping of genetic risk factors for chronic wasting disease in wild deer

**DOI:** 10.1111/eva.12003

**Published:** 2012-08-30

**Authors:** Tomomi Matsumoto, Michael D Samuel, Trent Bollinger, Margo Pybus, David W Coltman

**Affiliations:** 1 Department of Biological Sciences, University of AlbertaEdmonton, AB, Canada; 2 US Geological Survey, Wisconsin Cooperative Wildlife Research Unit, University of WisconsinMadison, WI, USA; 3 Department of Veterinary Pathology, Canadian Cooperative Wildlife Health Centre, University of SaskatchewanSaskatoon, SK, Canada; 4 Division of Fish and Wildlife, Alberta Sustainable Resource DevelopmentEdmonton, AB, Canada

**Keywords:** chronic wasting disease, genetic risk factor, linkage disequilibrium, microsatellite markers, mule deer, *NF1*, *PRNP*, white-tailed deer

## Abstract

Chronic wasting disease (CWD) is a fatal transmissible spongiform encephalopathy affecting North American cervids. We assessed the feasibility of association mapping CWD genetic risk factors in wild white-tailed deer (*Odocoileus virginianus*) and mule deer (*Odocoileus hemionus*) using a panel of bovine microsatellite markers from three homologous deer linkage groups predicted to contain candidate genes. These markers had a low cross-species amplification rate (27.9%) and showed weak linkage disequilibrium (<1 cM). Markers near the prion protein and the neurofibromin 1 (*NF1*) genes were suggestively associated with CWD status in white-tailed deer (*P* = 0.006) and mule deer (*P* = 0.02), respectively. This is the first time an association between the *NF1* region and CWD has been reported.

## Introduction

Chronic wasting disease (CWD) is a transmissible spongiform encephalopathy (TSE), or a prion disease, of North American cervids (family Cervidae), currently affecting both captive and wild elk (*Cervus elaphus*), mule deer (*Odocoileus hemionus*), white-tailed deer (*Odocoileus virginianus)*, and moose (*Alces alces*). TSEs are transmissible, fatal neurodegenerative disorders also commonly known in humans as Creutzfeldt–Jakob disease (CJD) and kuru, in sheep and goats as scrapie, and in cattle as bovine spongiform encephalopathy (BSE). TSE infectivity has been attributed to a misfolded conformer (PrP^Sc^) of the normal cellular prion protein (PrP^C^) (Prusiner 1989). While accumulation of PrP^Sc^ in the central nervous system is a key pathological feature, many underlying mechanisms of TSE pathogenesis including the normal physiological functions of PrP^C^ still remain elusive (reviewed in Westergard et al. 2007; Aguzzi et al. 2008). So far, no effective means of prevention or treatment have been developed, despite decades of extensive research (Aguzzi and Polymenidou 2004).

CWD is unique from other TSEs in its occurrence in the wild. Disease management in wild cervid populations has been severely impeded by efficient horizontal transmission of the disease agent, which has resulted in substantial economic losses to farming, gaming, and tourism industries (Bishop 2004; Seidl and Koontz 2004). Direct and indirect horizontal transmission is known to result from prion infectivity found in various tissues, fluids, and carcasses of infected animals (Miller and Williams 2003; Mathiason et al. 2006). Once in the environment, prions retain infectivity in soil for a prolonged period, aggravating the risk of exposure (Miller et al. 2004; Georgsson et al. 2006). This also raises an ecological concern for potential cross-species transmission to other sympatric mammals (Jennelle et al. 2009) and public health concern for the undetermined risk of human exposure to CWD through consumption of venison (Belay et al. 2004; Kong et al. 2005). Therefore, better understanding of CWD risk factors is a key to improved risk assessment and potential disease management applications. For example, genetic risk factors can be used to understand how heterogeneity in host susceptibility, infection rate, or incubation period affects CWD transmission dynamics (based on frequency of susceptible genotypes) and disease spread or predicts the future impact of CWD on deer populations. The characterization of genetic risk factors provides new insights into prion pathobiology, impacts of CWD on host fitness (Robinson et al. 2012a), and identifies potential targets for prophylactic treatment or therapy. It also provides potential targets for selective breeding to manipulate disease risk in captive deer or to understand natural selection in wild cervids (Robinson et al. 2012a).

Polymorphisms in the prion protein gene (*PRNP*) are known to influence host susceptibility. While some genotypes in humans (Aguzzi 2006) and sheep (Hunter 2007) confer resistance to TSEs, susceptible *PRNP* polymorphisms generally predominate in wild deer populations (see review in Robinson et al. 2012b). Furthermore, the fact that *PRNP* explains only part of the genetic variance in TSEs (Diaz et al. 2005; Lloyd and Collinge 2005) and that other quantitative trait loci (QTL) and candidate genes have been discovered (e.g., Stephenson et al. 2000; Lloyd et al. 2001; Moreno et al. 2003; Zhang et al. 2004; Mead et al. 2009) suggest there are multiple underlying genetic risk factors. The only other candidate gene studied so far, complement component C1q, was weakly associated with susceptibility in wild white-tailed deer (Blanchong et al. 2009). Some other promising candidate risk factors include *IL1B* and *1L1RN,* which are members of the interleukin-1 (IL-1) gene family encoding IL-1**β** and its receptor antagonist IL-1RA, respectively. They are mediators in the inflammatory response and risk factors for Alzheimer's disease (Sciacca et al. 2003; Licastro et al. 2004) that may also have functional and positional (QTL) links to TSE (Schultz et al. 2004; Marcos-Carcavilla et al. 2007). Neurofibromin 1 (*NF1*) is a tumor suppressor gene responsible for inherited neurofibromatosis type 1 disorder (Trovó-Marqui and Tajara 2006) that is also a strong positional candidate for TSE (Stephenson et al. 2000; Lloyd et al. 2001, 2002; Geldermann et al. 2006).

No QTL mapping studies for CWD in deer have been conducted to date, likely because the resources required to conduct a mapping study in deer are not available. QTL studies require a genetic linkage map and a genotyped population of known pedigree in which the trait of interest is segregating. However, it also possible to detect genetic risk factors for complex diseases using association mapping approaches in open populations (Kruglyak 1999; Hirschhorn and Daly 2005; McCarthy et al. 2008). Association mapping relies on detecting correlations between genotypes and the phenotype of interest that are generated by linkage disequilibrium (LD) across a sample of unrelated individuals. Whole genome association (WGA) studies of human TSEs and BSE using extensive single-nucleotide polymorphism (SNP) chips have recently revealed highly localized genomic regions associated with TSE susceptibility (Mead et al. 2009; Murdoch et al. 2010). WGA studies of CWD have not been possible, given the lack of genomic resources for deer. However, the genetic map of the subfamily Cervinae established by Slate et al. (2002), coupled with chromosomal homology with cattle, sheep, and humans, provides a useful comparative framework to conduct coarse-scale association mapping in deer using cross-amplified microsatellites from predicted linkage groups. Based on >50% cross-amplification rates for bovine microsatellite markers in cervids (Kühn et al. 1996; Slate et al. 1998) and the density of the bovine genetic map (∼3800 markers), there is potential to produce high-resolution linkage maps in deer using cross-amplification.

Experimental studies of CWD are costly and challenging to conduct because they require large numbers of animals to be maintained under controlled conditions for long periods of time, given the long incubation period for CWD. Furthermore, artificial interactions between wild deer in experimental studies do not accurately represent disease transmission in free-living deer. We therefore utilized wild white-tailed deer and mule deer sampled from CWD-affected areas in Wisconsin and Saskatchewan, respectively (Joly et al. 2003; Kahn et al. 2004). These well-studied areas (Cullingham et al. 2011a; Cullingham et al. 2011b; Rogers et al. 2011; Robinson et al. 2012a) are also subject to extensive surveillance and testing and are therefore logistically feasible populations in which to conduct our association study.

Our goal was to assess the feasibility of association mapping and to identify novel CWD genetic risk factors in wild deer using a matched case–control study design (Kleinbaum et al. 1982) that considered as many confounding risk factors as possible, including spatial locations, age, and sex (Miller and Conner 2005; Grear et al. 2006; Joly et al. 2006; Blanchong et al. 2009; Osnas et al. 2009).We first conducted cross-species amplification of microsatellite markers from a high-density genetic map of cattle. We then estimated LD between markers to determine whether this method would yield sufficient marker density for association mapping. Finally, we tested for association of CWD status with three candidate regions: *PRNP* on linkage group (LG) 23, *IL1B* and *1L1RN* on LG11, and *NF1* on LG5.

## Materials and Methods

### Samples and DNA extraction

We analyzed two sets of white-tailed deer and one set of mule deer DNA samples. The first set of CWD-negative white-tailed deer (*N* = 184) was used to optimize bovine microsatellite markers for amplification and subject to LD and population structure analyses. The second, separate set of white-tailed deer samples (*N* = 192) were matched case–controls selected for association testing. For mule deer, a single matched case–control set of samples (*N* = 192) was used for the LD and population analyses and the association testing. In both sets of matched case–controls, one half of the animals (96) were CWD positive and the other half (96) were CWD negative.

The CWD-negative white-tailed deer samples were collected across the CWD management zone in Wisconsin during the 2002 hunting surveillance season (Grear et al. 2006). The matched case–control samples were obtained from the core epidemic area (∼303 km^2^) where CWD prevalence is highest (Joly et al. 2006). The Wisconsin Veterinary Diagnostic Laboratory conducted the CWD testing on retropharyngeal lymph nodes and brain stem (obex) tissue by immunohistochemistry or enzyme-linked immunosorbent assay (ELISA). Saskatchewan mule deer samples were provided through hunter submission, and sampling of retropharyngeal lymph nodes was by the University of Saskatchewan and the Canadian Cooperative Wildlife Health Centre during the provincial disease control effort from 2001 to 2007. Most of the mule deer samples were collected from the southern CWD range along the South Saskatchewan River Valley, a few came from the northern CWD range along the North Saskatchewan River (Wilson et al. 2009) and were excluded from association testing (*N* = 18). CWD testing was performed by standard immunohistochemistry techniques using tonsil or retropharyngeal lymph node tissues.

We individually matched case–control samples using three criteria known to influence CWD prevalence in the wild: location, sex, and age. The matched white-tailed deer samples (*N* = 192) consisted of 96 female pairs ≥2 years old. For each case sample, a control from the same ∼2.6 km^2^ section (defined by the Public Land Survey System of Wisconsin) with matched age was selected. When an exact match was unavailable, we used one with similar age or from an adjacent section. In Saskatchewan, availability of samples was more limited, thus matching was less stringent. The matched mule deer sample consisted 47 male pairs and 40 female pairs (*N* = 174) all ≥1.5 years old. For each case sample, a control with the same or similar age and closest geographic location was selected. The mean (±standard deviation) distance between pairs was 14.9 (±12.1) km, ranging from 1.9 to 57.2 km.

DNA was extracted via a standard phenol–chloroform method from frozen skeletal muscle tissue (CWD-negative white-tailed deer), frozen retropharyngeal lymph nodes (matched case–control white-tailed deer), and ethanol-fixed tissues (matched case–control mule deer) all stored at −20°C. Approximately 0.1 g of tissue was incubated in 600 μL of extraction buffer (20 mm Tris–chloride, pH 8.0; 20 mm EDTA, pH 8.0; 20 μL/mL RNase A, DNase-free; 0.1% SDS) for 20 min at 65–67°C and digested overnight at 50–52°C following the addition of 20 μL of 20 mg/mL Proteinase K. The same amount of Proteinase K was added and incubated for additional 1–2 h at 50–52°C. Three rounds of organic extraction were performed using equal volumes of phenol, 1:1 mixture of phenol/chloroform, and chloroform. DNA was precipitated and washed twice: first by adding 1/10 volume of 3 m sodium acetate and 2.25 volumes of 95% ethanol and second with 500 μL of 70% ethanol. Extracted DNA was eluted with 200 μL of miliQ H_2_O, quantified with Nanodrop™ 2000 (Thermo Fisher Scientific Inc., Wilmington, DE, USA), and standardized at ∼20 ng/μL.

### Cross-species amplification

We selected three red deer LGs and four corresponding cattle homologues: LG 23 (Bta 13), LG 11 (Bta 11), and LG 5 (Bta 17 and 19, Robertsonian-fused). The selection was based on two criteria: (i) predicted assignment of candidate genes and QTL previously identified for other TSEs and (ii) high degree of conservation with cattle homologues established by Slate et al. (2002). The three LGs were predicted to harbor candidate regions for *PRNP*, *IL1B* and *1L1RN*, and *NF1* (Ihara et al. 2004), respectively, along with predicted intervals corresponding to QTL for scrapie and BSE mapped to the mouse and cattle genomes (Stephenson et al. 2000; Lloyd et al. 2001, 2002; Marcos-Carcavilla et al. 2007). We selected 215 microsatellite markers from the high-density microsatellite map (Ihara et al. 2004) at an interval of ∼2.5 cM with additional markers in the candidate regions. Bovine primer sequences were obtained from Ihara et al. (2004) and synthesized using the M13 florescent primer labeling system (Schuelke 2000).

The bovine primers were screened for polymerase chain reaction (PCR) amplification using a panel of six to seven white-tailed deer along with positive control cattle DNA and a negative control (milliQ H_2_O). Screening PCR was performed in a total volume of 15 μL, consisting of ∼50 ng of template DNA, 1× PCR buffer (10 mm Tris–Cl, pH 8.8; 50 mm KCl; 0.1% Triton X-100; 0.16 mg/mL bovine serum albumin, nuclease free), 1.9 mm MgCl_2_, 0.2 mm each dNTPs, marker-specific primers (0.04 μm M13-modified forward primer and 0.16 μm reverse primer), 0.16 μm dye-labeled M13 primer, and 0.5 U Taq polymerase isolated as in Engelke et al. (1990). Amplification was performed on Mastercycler® ep gradient (Eppendorf, Hamburg, Germany) prewarmed to 94°C denaturation temperature, with the following conditions: 1-min initial denaturation at 94°C; three cycles of 30 s at 94°C, 20 s at 52°C, 5 s at 72°C; 30 cycles of 15 s at 94°C, 20 s at 52°C, 5 s at 72°C; and 15-min final extension at 72°C. Amplified fragments were resolved with GeneScan™ 500 LIZ® Size Standard (Applied Biosystems) on a 48-capillary 3730 DNA analyzer (Applied Biosystems), and resulting electropherograms were inspected for amplification of microsatellite peaks using GeneMapper® Software v4.0 (Applied Biosystems, Foster City, CA, USA). We first genotyped the CWD-negative white-tailed deer samples using bovine primers that successfully amplified polymorphic loci on the screening panel (screening result for each marker is listed in Table S1). We then used markers that yielded reliable genotypes across the CWD-negative white-tailed deer samples to genotype the matched case–control mule deer and white-tailed deer samples. Optimization of PCR conditions was done by adjusting MgCl_2_ concentrations (2.5 or 3.0 mm) and/or annealing temperature (Ta) with touchdown protocols (Korbie and Mattick 2008).

Genotype data were compiled, and the number of alleles, observed (*H*_O_) and expected (*H*_E_) heterozygosity for each locus, was calculated in the Excel Microsatellite Toolkit (Park 2001). Genepop v4.0 (Rousset 2008) was used to calculate, for each locus, the exact probabilities of Hardy–Weinberg equilibrium (HWE) and *F*_IS_ by Weir and Cockerham (1984)'s estimate. Because we observed slightly positive mean *F*_IS_ in all three sets of samples, possibly due to null alleles or errors, we identified outliers in the *F*_IS_ frequency distribution and excluded them from the following analyses. Supplementary materials on individual marker results and PCR conditions can be found in Tables S2 and S3.

### LD and population structure analyses

Because of high polymorphism and small sample sizes, instead of estimating haplotype phases, we opted for genotype-based LD composite measures (Weir 1979). For each pair of loci, the squared correlation coefficient (composite *r*^2^) was obtained using the program developed by Zaykin et al. (2008) and plotted against predicted intermarker distances for each LG. An exponential decay regression of the form *y* = *y*_0_ + *a*e^−*bx*^ was fitted to each plot. Here, the maximum value at 0 cM is estimated by *y*_0_ + *a*, and the minimum or background value is estimated by *y*_0_. The rate of decline over distance, denoted as *x*_1/2_, was estimated at the point where *r*^2^ declined to the midpoint between the maximum and minimum *r*^2^, and was calculated from the regression as 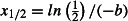.

Statistically significant LD between each pair of loci was assessed by the probability of genotypic association in Genepop v4.0. LD between syntenic markers was quantified as the proportion of marker pairs in each predicted intermarker interval that was in significant LD at the nominal level (*P* = 0.05). Likewise, proportions of nonsyntenic marker pairs in significant LD represented the background levels of LD not because of physical proximity. Deviations from the expected level (α = 0.05) were assessed by chi-square test. We tested whether the proportions of significant LD for syntenic marker intervals were significantly different from the nonsyntenic background levels using a *Z*-test in Sigma Plot v11.0 (Systat Software Inc, San Jose, CA, USA). The Bonferroni correction was used to account for multiple testing. We repeated significance testing using data sets that pooled all rare alleles (frequency < 0.05) at each locus whenever possible. This was to safeguard against a potential loss of power and statistical error because of the high levels of allelic diversity observed in both species.

We tested for potentially confounding population structure within our samples using a *F*_IS_-based permutation test in Fstat v2.9.3 (Goudet 1995) and a Bayesian clustering method implemented in Structure v2.2.3 (Pritchard et al. 2000). Only loci that were not in significant LD (*P* < 0.05) were used in these analyses, resulting in 30 loci in white-tailed deer and 22 loci in mule deer. A series of models assuming *K* = 1–10 subpopulations with admixture and correlated allele frequencies were run, each with five replicates and a run length of 100 000 steps for burn-in and 100 000 steps for a parameter estimation.

### Association testing

We used conditional logistic regression (Kleinbaum et al. 1982) to test for CWD association in the matched case–control data using SAS v9.2 (SAS Institute Inc., Cary, NC, USA). For each locus, the probability of infection (*p*) was modeled using presence/absence (*x*) of each allele (1, 2, …, *k*) as exposure variables, hence the model took the form logit(*p*) = α + *x*β_1_ + *x*β_2_ + … *x*β_*k*_. Rare alleles (<0.05) were pooled to maintain statistical power. Significance was determined by likelihood ratio tests, and *P*-values (negative log transformed) were plotted against predicted marker positions inferred from the bovine map (Ihara et al. 2004) using MapChart v2.1 (Voorrips 2002). Bonferroni correction was used to obtain LG-wide and study-wide significance levels.

## Results

### Cross-species amplification and marker density

A total of 70 markers of 215 were successfully genotyped in the white-tailed deer CWD-negative sample set. Of these 70 markers, 45 were successfully genotyped in the matched mule deer case–control sample (Tables S2 and S3). There were ten outlying, excluded loci (*F*_IS_ > 0.15) in white-tailed deer and three (*F*_IS_ > 0.16) in mule deer. The white-tailed deer and mule deer marker panels used in the LD analyses thus consisted of 60 (27.9% of the initial markers screened) and 42 loci (60.0% of the markers typed in white-tailed deer), respectively. The matched case–control white-tailed deer sample was genotyped using the 60 markers on the white-tailed deer panel, of which four (DIK4520 on LG23; VH98, BM9138, and X82261 on LG5) failed to amplify across the sample set (>95%) and thus were excluded from the analysis. Three additional outlying loci (*F*_IS_ > 0.1 and deviating from HWE) (UMBTL184 and BM3501 on LG11; DIK2200 on LG5) were excluded from the following analysis. Consequently, 53 markers were tested for association in white-tailed deer and 42 markers in mule deer.

Both species were highly diverse, and the mean (±SD) number of alleles was 10.7 (±6.3) and 9.8 (±5.0) in the CWD-negative and matched case–control white-tailed deer samples, respectively, and 7.8 (±3.5) in mule deer. The mean *H*_O_ was 0.70 (±0.25) and 0.69 (±0.23) in white-tailed deer and 0.64 (±0.19) in mule deer. The mean *H*_E_ was not different than observed in both species: 0.72 (±0.23) and 0.70 (±0.23) in white-tailed deer and 0.65 (±0.19) in mule deer. Small but positive mean *F*_IS_ was observed in both species: 0.029 (±0.042) and 0.019 (±0.035) in white-tailed deer and 0.019 (±0.049) in mule deer.

**Figure 1:**
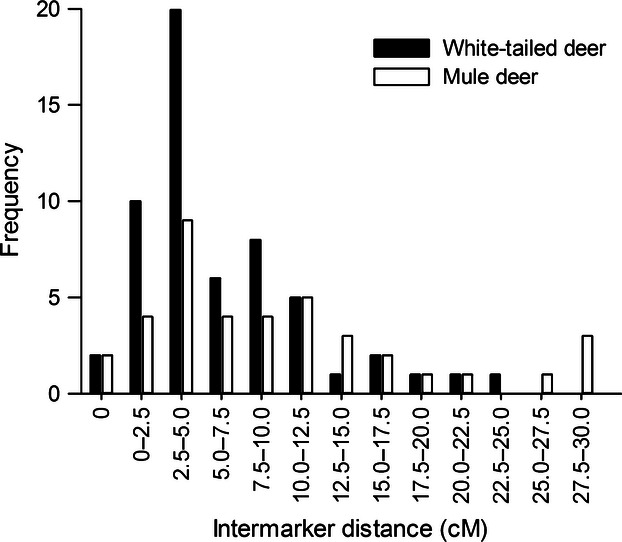
Frequency distribution of predicted intermarker distances (cM) for the deer microsatellite marker panels (red deer LG23, 11, and 5). The panels shown are 60 markers genotyped in the chronic wasting disease-negative white-tailed deer and 42 markers genotyped in the matched mule deer samples. Marker positions were inferred from the bovine map (Ihara et al. 2004).

The marker panels had a mean predicted intermarker interval of 6.3 (±5.3) cM in white-tailed deer (60 markers) and 9.8 (±8.1) cM in mule deer (42 markers). Predicted intervals were highly variable, ranging from 0 to 24.68 cM in white-tailed deer and 0–29.49 cM in mule deer (Fig. 1).

### LD and population structure

**Figure 2:**
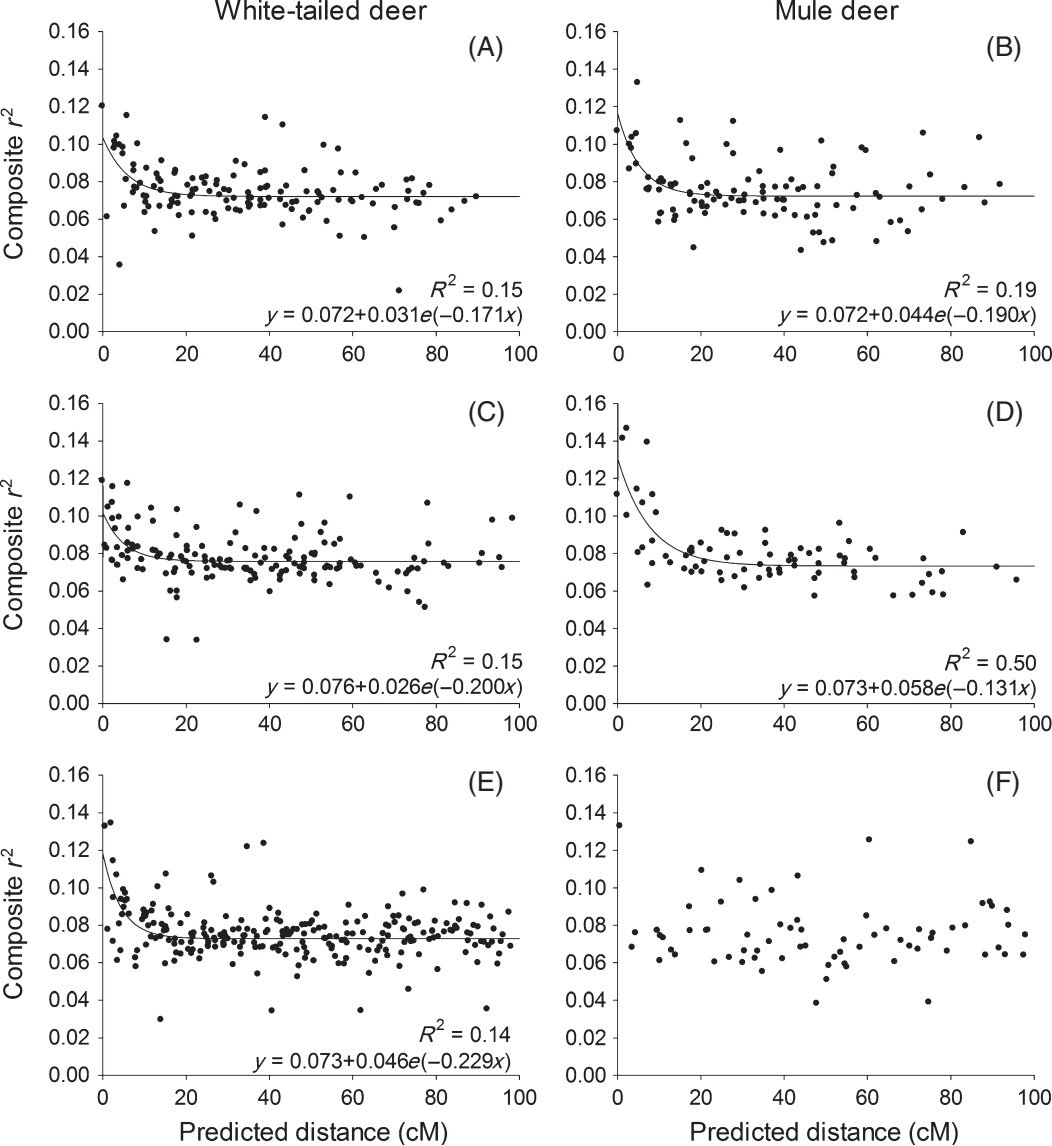
Composite *r*^2^ between syntenic marker pairs as a function of predicted marker distance (cM) for red deer LGs 23 (A, B), 11 (C, D), and 5 (E, F) in white-tailed deer and mule deer. Marker distances were inferred from the bovine map (Ihara et al. 2004). Exponential decay regression lines (*y* = *y*_0_ + *a*e^−*bx*^) were fitted. All coefficients tested significant (*P* < 0.05) except for LG5 in mule deer (F).

Both species exhibited weak correlation of genotypes between pairs of markers across all LGs (*r*^2^ < 0.15; Fig. 2). An exponential decline in *r*^2^ with predicted marker distance was observed in most LGs with the exception of LG5 in mule deer (Fig. 2F). However, the regressions often explained a small fraction of the variation in *r*^2^ (*R*^2^ < 0.2). While the background levels of *r*^2^ were consistent across all LGs, ranging from 0.072 to 0.076, the patterns of decline in *r*^2^ varied among LGs.

**Figure 3:**
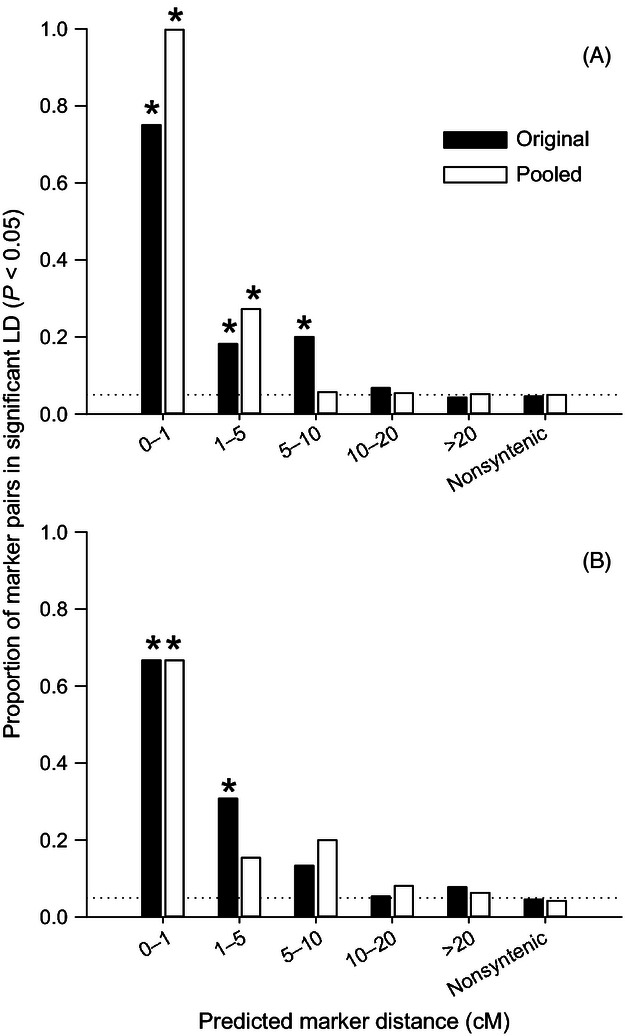
Proportions of marker pairs in significant linkage disequilibrium (LD) (*P* < 0.05) summarized by predicted marker intervals (cM) in white-tailed deer (A) and mule deer (B) tested using both original genotype and pooled genotype data (rare alleles with frequency < 0.05 were pooled). Dotted lines indicate the type I error rate (α = 0.05). Proportions of significant LD in nonsyntenic marker pairs were statistically equivalent to the type I error rate in both species using both data sets. *Significant difference from the nonsyntenic proportions after the Bonferroni correction.

The background levels of LD, measured by the proportions of nonsyntenic marker pairs in significant LD (*P* < 0.05), were equivalent to the type I error rate (α = 0.05) in both species in both the original and rare-frequency allele pooled data sets (Fig. 3) (the original and the pooled data sets, respectively: 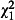 = 0.37, *P* = 0.54 and 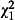 = 0, *P* = 1 in white-tailed deer; 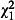 = 0.13, *P* = 0.72 and 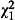 = 0.54, *P* = 0.46 in mule deer). Most marker pairs within 1 cM were in significant LD, with the proportions in significant LD above the background level in both species in both data sets (Fig. 3) (*Z* = 5.40, *P* < 0.001 and *Z* = 7.34, *P* < 0.001 in white-tailed deer; *Z* = 3.62, *P* < 0.001 and *Z* = 3.78, *P* < 0.001 in mule deer). LD approached background levels in the 10–20 cM range. In white-tailed deer, statistically higher LD extended 5–10 cM in the original data set (1–5 cM: *Z* = 3.15, *P* = 0.002; 5–10 cM: *Z* = 3.73, *P* < 0.001) and only 1–5 cM in the pooled data set (1–5 cM: *Z* = 5.11, *P* < 0.001; 5–10 cM: *Z* = −0.20; *P* = 0.84) (Fig. 3A). In mule deer, elevated proportions were also observed up to 5–10 cM in both data sets, but only the 1–5 cM proportion in the original data set was statistically significant (1–5 cM: *Z* = 3.58, *P* < 0.001; 5–10 cM: *Z* = 0.95; *P* = 0.34) but not in the pooled data set (1–5 cM: *Z* = 1.24, *P* = 0.22; 5–10 cM: *Z* = 2.24; *P* = 0.03) (Fig. 3B).

**Figure 4:**
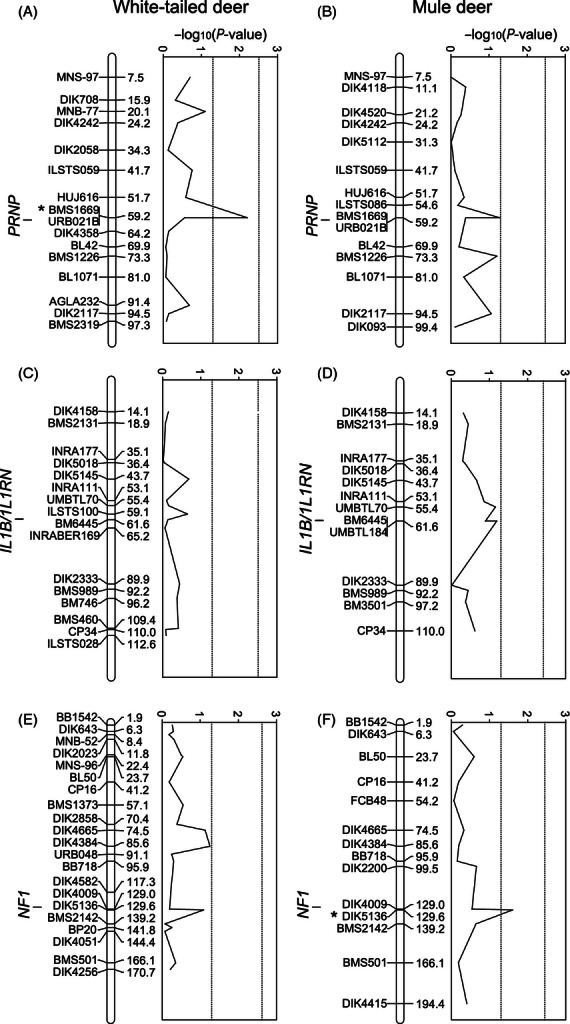
Microsatellite marker panels and probability of marker associations with chronic wasting disease in white-tailed deer and mule deer matched case–control samples. Predicted positions (cM) of the markers and candidate genes for red deer LGs 23 (A, B), 11 (C, D), and 5 (E, F) were inferred from the bovine map (Ihara et al. 2004). Vertical lines show nominal and LG-wide significance levels, respectively. Markers above the nominal significance are indicated with*.

We observed highly significant LD (*P* ≪ 0.001) between markers almost exclusively within a predicted interval of 1 cM, and these were mostly pairs co-located in the candidate regions. In the matched case–control white-tailed deer and mule deer samples, the marker pair BMS1669 and URB021B in the *PRNP* region (Fig. 4A,B) was in strong LD in white-tailed deer (*P* < 0.001) and weakly linked in mule deer (*P* = 0.08). In the *IL1B/IL1RN* region, the marker pair BM6445 and UMBTL184 (Fig. 4C,D) was in strong LD in mule deer (*P* < 0.001). In the *NF1* region, the marker pair DIK4009 and DIK5136 (Fig. 4E,F) predicted to be within 0.6 cM of each other was in strong LD in both white-tailed deer (*P* < 0.001) and mule deer (*P* < 0.01).

We observed a small positive mean *F*_IS_ that was statistically significant (*P* = 0.002 in white-tailed deer and *P* = 0.01 in mule deer), indicating small degrees of nonrandom mating within each population. However, no distinct subpopulation was detected in the structure analyses as the posterior probability (ln Pr(*X*|*K*)) was the highest for *K* = 1 in both species.

### Association testing

No markers tested in white-tailed deer and mule deer matched case–control samples, respectively, were significantly associated with CWD status at LG-wide (α = 0.002–0.004) or study-wide significance levels (α = 0.0009–0.001) (Fig. 4). Two markers showed significant association at the nominal level (α = 0.05): BMS1669 (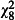 = 21.4, *P* = 0.006) on LG23 in white-tailed deer (Fig. 4A) and DIK5136 (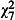 = 16.03, *P* = 0.02) on LG5 in mule deer (Fig. 4F). Both of these markers were predicted to be near (<1 cM) the candidate genes, BMS1669 near *PRNP* and DIK5136 near *NF1*, and each was in strong LD with another nearby marker (≤0.6 cM) in the same region. They were also nearly significantly associated with CWD in the other species (BMS1669 in mule deer, 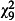 = 16.78, *P* = 0.05, Fig. 4B; DIK5136 in white-tailed deer, 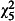 = 9.79, *P* = 0.08, Fig. 4E). If we combined the statistical significance of markers in common between the two population analyses using Fisher's (1954) method, the association of BMS1669 was statistically significant at the linkage group level (*P* = 0.0028, Bonferroni correction for 10 markers in LG 23 α = 0.005), and the association of DIK5136 was nominally statistically significant (*P* = 0.015). Markers located near *IL1B* and *IL1RN* were nonsignificant in both species (Fig. 4C, D); although one of them (UMBTL184) was nearly significant in mule deer (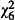 = 11.89, *P* = 0.06, Fig. 4D), this marker was not on the white-tailed deer panel because of high *F*_IS_. The detailed results of the association tests for each marker are listed in supporting material Tables S3, S4 and S5.

## Discussion

Alleles at two loci predicted to reside in candidate gene regions, BMS1669 in white-tailed deer (candidate gene PRNP; Fig. 4A) and DIK5136 in mule deer (candidate gene NF1; Fig. 4F), suggested association with CWD at the nominal level. The same markers also showed nearly significant association (*P* = 0.05 and *P* = 0.08, respectively) in the other species (Fig. 4B, E), which supports these associations. Furthermore, *PRNP* is a known risk factor for CWD (e.g., Wilson et al. 2009; Robinson et al. 2012a), providing validation of our ability to detect association through linked markers, despite weak population LD. We also did not find an excess number of significant associations at other loci within the same linkage groups, indicating our results are unlikely to be spurious associations because of cryptic genetic substructure.

Our results identify two CWD genetic risk factors that merit further research and provide a potential key to improved risk assessment and disease management in wild cervids. For example, information on the relative abundance or spatial genetic variation of PRNP and NF1 genetic variants can be incorporated into models of CWD spread and impact on deer populations. Further research on NF1 might provide new insights into prion pathobiology, identify a potential target for prophylactic treatment, or identify a selective breeding target to manipulate disease risk in captive deer.

The detection of the significant association near *PRNP* corroborates its role as an important risk factor for CWD. Across species and regions, however, ‘resistance’-associated alleles predispose animals to longer incubation periods (O'Rourke et al. 2004; Hamir et al. 2006) and do not prevent infection (O'Rourke et al. 2004; Wilson et al. 2009; Robinson et al. 2012b). The associations at BMS1669 (∼0.6 Mb from PRNP; Fig. 4A,B) likely reflect the effects of linked *PRNP* alleles. While it is also possible that causative polymorphisms outside the *PRNP* coding regions affect expression of *PRNP* or other linked genes (Perucchini et al. 2008), the fact that the next closest marker in our panel URB021B (Fig. 4A,B), which is ∼1.4 Mb from PRNP in the bovine reference genome (NC_007311.4; Elsik et al. 2009), was not associated with CWD status supports the idea that the effect is attributable to *PRNP*.

In addition to *PRNP*, experimental QTL studies have identified other genetic risk factors but often suffered inconsistent results because of the use of different lines of inbred mice and prion strains (e.g., Stephenson et al. 2000; Lloyd et al. 2001; Manolakou et al. 2001). The *NF1* region, however, has been implicated by multiple studies. It is contained within QTL on mouse chromosome 11 associated with experimental scrapie (Stephenson et al. 2000; Lloyd et al. 2001) and BSE incubation periods (Lloyd et al. 2002). Zhang et al. (2004) also suggested a QTL on cattle chromosome 19 in naturally BSE-infected cattle. Furthermore, Geldermann et al. (2006) found significant associations at two markers surrounding the *NF1* region (∼4 cM apart in Ihara et al. 2004 map) in case–control BSE breeds. Thus, the association we found in mule deer (Fig. 4F), the first evidence for a CWD association, supports the idea that the *NF1* region likely contains risk loci. While it is critical to note that a number of other studies did not detect QTL or associations in this region (Manolakou et al. 2001; Hernandez-Sanchez et al. 2002; Moreno et al. 2003; Murdoch et al. 2010), the difficulty of replicating QTL and association results and the fact that the *PRNP* region was not always found significant by the previous mapping studies (e.g., Hernandez-Sanchez et al. 2002; Zhang et al. 2004; Murdoch et al. 2010) suggest that the *NF1* region merits further investigation.

Identification of the actual risk genes from previously identified QTL regions is hampered by the vast number of genes harbored within a QTL region (Hirschhorn and Daly 2005). With the limited LD (<1 cM) in white-tailed deer and mule deer, it may be possible to narrow the candidates in the *NF1* region. Based on the bovine reference genome sequence (NC_007317.4; Elsik et al. 2009), the other nonsignificant tag marker (DIK4009) is predicted to be located closer (∼0.8 Mb) to the *NF1* locus than DIK5136 (∼1.6 Mb). Given that the markers themselves were in significant LD in both deer species, the actual risk gene may be located closer to DIK5136 and not *NF1*. While no clear connection between *NF1* and TSEs has been suggested, the region surrounding (∼0.5 Mb) DIK5136 has a high density of genes including some that are potentially relevant to TSE (e.g., genes encoding: microRNAs, *MIR451* and *MIR144*; a lipid raft protein flotillin-2; a glycolytic enzyme aldolase-C). For example, microRNAs (miRNAs) regulate gene expression via RNA silencing, and dysfunctional regulation by some miRNAs was recently implicated in accumulation of amyloid proteins in Alzheimer's disease and TSEs (reviewed in Provost 2010). Also, alteration in lipid raft constituents has been hypothesized to play a role in a variety of neurodegenerative diseases including TSEs (reviewed in Schengrund 2010). PrP^c^ is a glycosylphosphatidylinositol (GPI)-anchored protein associated with lipid rafts and suspected to interact with fotillin-2 and flotillin-1 in various signaling pathways (Schengrund 2010; Solis et al. 2010). Similarly, aldolase-C was identified as an interactor of PrP^c^ (Strom et al. 2006), and its transcripts were found to be overexpressed in BSE-infected mice (Dandoy-Dron et al. 2000). Thus, targeted investigation of these and other genes close to DIK5136 may be an effective starting point for mining the *NF1* region.

The failure to detect significant association near *IL1B* and *IL1RN* (Fig. 4C,D) suggests these genes unlikely contribute strong risk, especially as the tag markers were predicted to be within 0.1–0.2 Mb (NC_007309.4; Elsik et al. 2009). However, one of the markers (UMBTL184) was nearly significantly associated (*P* = 0.09) in mule deer (Fig. 4D) and was not tested in white-tailed deer because of null alleles. This region may need to be re-examined, given other evidence linking these genes to TSEs (Burwinkel et al. 2004; Schultz et al. 2004; Marcos-Carcavilla et al. 2007).

While these results are potentially valuable in their application to CWD management and future understanding of prion pathobiology, our results also exemplified the difficulty in detecting highly significant association at LG-wide or study-wide levels in wild populations using microsatellites. While the matched case–control design is efficient (Kleinbaum et al. 1982), our sample sizes were small relative to the size of the marker panels and the high allelic diversity of the microsatellite loci. Small sample size and low marker density limit our power to detect risk-associated alleles, especially those with moderate effects on CWD prevalence.

Our polymorphic amplification rate of bovine markers in white-tailed deer (27.9%) was lower than previous estimates (>50% by Kühn et al. 1996; Slate et al. 1998). This may be due to differences in laboratory protocols, levels of optimization effort, stringency of criteria for success, sample sizes, and biased selection of well-tested markers in previous studies. Our amplification rate is probably representative for markers from the high-density bovine microsatellite map. It also seems to be in agreement with the large evolutionary distance between the families Bovidae and Cervidae with the estimated divergence time of about 30 million years ago (MYA) (Hassanin and Douzery 2003; Fernandez and Vrba 2005). The white-tailed deer marker panel, containing 60 loci across three predicted LGs, had a mean predicted interval of 6.3 cM, which is two to three times the marker density of the current red deer genetic map (Slate et al. 2002) and only slightly higher than the first-generation low-density bovine microsatellite maps (Bishop et al. 1994; Ma et al. 1996). There is, therefore, the potential to develop a higher resolution map with the screening of additional bovine markers. However, quite a large number of markers will be required, given the weak levels of LD we found in wild deer.

We observed higher than background levels of LD to extend beyond 1 cM and potentially to 5 cM, based on the composite *r*^2^ (Fig. 2) and the proportions of marker pairs in significant LD (Fig. 3). These levels of association are, however, expected to be too weak to be useful for association mapping. This is because at 1–5 cM, only ∼20% of the marker pairs were in significant LD at the nominal level (*P* < 0.05) with few of them highly significant (*P* ≪ 0.001). The highly significant associations were largely restricted to predicted intervals <1 cM, where there were only four white-tailed deer and three mule deer marker pairs. This meant that we are unable to characterize the extent of useful LD at a finer scale (≪1 cM) and may have led to the low composite *r*^2^ values (<0.15) even at 0 cM predicted distance (Fig. 2). Even our most closely linked markers are too sparsely located for association mapping in these populations.

We should note that composite LD measured from microsatellite genotypes has statistical properties that are not as easily interpretable as standard measures for SNP-based haplotype data. For example, while multi-allelic *r*^2^ has proven to be more robust to small sample sizes and allele frequencies, particularly rare alleles (Ardlie et al. 2002; Weiss and Clark 2002; Zhao et al. 2005), none of the formulae for extending common measures (*D'*, *r*^2^, and *χ*^2^) to multi-allelic situations are independent of allele frequencies (Hedrick 1987; Zhao et al. 2005). Our analysis also revealed a reduced range of statistically significant LD in both species when rare alleles were pooled (Fig. 3), suggesting a risk of overestimating LD by not accounting for rare alleles. Moreover, the interpretation of LD from genotype-based measures is confounded by a departure from HWE (Weir 1979) and diminished power compared with cases where haplotype information is available (Pritchard and Przeworski 2001; Slatkin 2008). Therefore, the low levels of composite *r*^2^ in these populations (Fig. 2) may be conservative. Unfortunately, haplotype frequency estimation or phase reconstruction procedures were impractical for our data because of the large number of alleles and small sample sizes.

The weak overall LD (≪1 cM) means that dense SNP panels are likely required to characterize patterns of short-range LD. Such weak levels of LD are found in humans (Ardlie et al. 2002) and some open, outbreeding wildlife populations (Laurie et al. 2007; Gray et al. 2009). Livestock populations typically show higher levels of LD because of their historical demography and artificial selection (Farnir et al. 2000; Sutter et al. 2004; Amaral et al. 2008). While weak LD means that large numbers of markers are required for efficient association testing, it also suggests that significant associations are likely to be relatively close to the causative mutation. This is in contrast to livestock populations, where association testing can detect significant associations a long way from the causative mutation because of long-range LD. Among wild animals, long-range LD has been found in inbred wolf populations (Gray et al. 2009), collared flycatcher (*Ficedula albicollis*) (Backström et al. 2006), bighorn sheep (*O. canadensis*) (Miller et al. 2011), and Siberian jays (*Perisoreus infaustus*) (Li and Merila 2010). Weak LD in our wild deer populations contrasts with the strong LD found in the red deer (Slate and Pemberton 2007), likely due to admixture from the recent introduction of a reproductively highly successful male into the island population. The absence of significant LD between distant (>5–10 cM) and nonsyntenic marker pairs in our open study populations (Fig. 3) is a likely characteristic of much of the species range. Both species are abundant habitat generalists with semi-continental distributions in North America, consistently high genetic diversity, and high dispersal capability (e.g., Van Den Bussche et al. 2002; DeYoung et al. 2003; Latch et al. 2009).

Our work identifies several avenues of future research on CWD in wild cervids, ideally harnessing next-generation sequencing technology to develop high-density SNP panels to finely map the associations near candidate regions such as PRNP and NF1. The development of genomic resources for wildlife species is still in the very early stages. However, SNP development and genotyping strategies that use restriction enzyme digestion to reduce the complexity of the target genome can be applied to nonmodel species using next-generation sequencing (Davey et al. 2011). Pyrosequencing of a reduced representation white-tailed deer genome recently yielded a complete mitochondrial genome sequence and ∼10 000 putative genomic SNPs (Seabury et al. 2011). These approaches provide new avenues for future genomic and evolutionary applications in ecologically important wildlife species that lack suitable reference genomes.

## Data archiving statement

Data deposited in the Dryad repository: doi:10.5061/dryad.qf2vm.

## Supporting Information

**Table S1.** Amplification results of 215 bovine microsatellite markers in white-tailed deer screening panel (*N* = 7).

**Table S2.** PCR conditions, diversity statistics, and potential genotyping issues of 70 bovine microsatellites in white-tailed deer (*N* = 184) from Wisconsin.

**Table S3.** Microsatellite marker panel, PCR conditions, diversity statistics, and the results of CWD association tests in white-tailed deer (*N* = 192) and mule deer (*N* = 174) matched samples.

**Table S4.** CWD case–control association test results by conditional regression for 53 microsatellite markers in white-tailed deer from Wisconsin.

**Table S5.** CWD case–control association test results by conditional regression for 42 microsatellite markers in mule deer from Saskatchewan.
